# Supplementary material for: Glycolipid MPIase is essential for the TAT (twin-arginine translocation) pathway
Source: eLife. 2026 Nov 2;15:RP112170. doi: 10.7554/eLife.112170 (PMC13727073; doi:10.7554/eLife.112170)
Supplement: Supplementary file 1 [file elife-112170-fig1-data1.zip › Figure 1-source data 1/Figure 1-source data 1.pdf]

Fig.1A

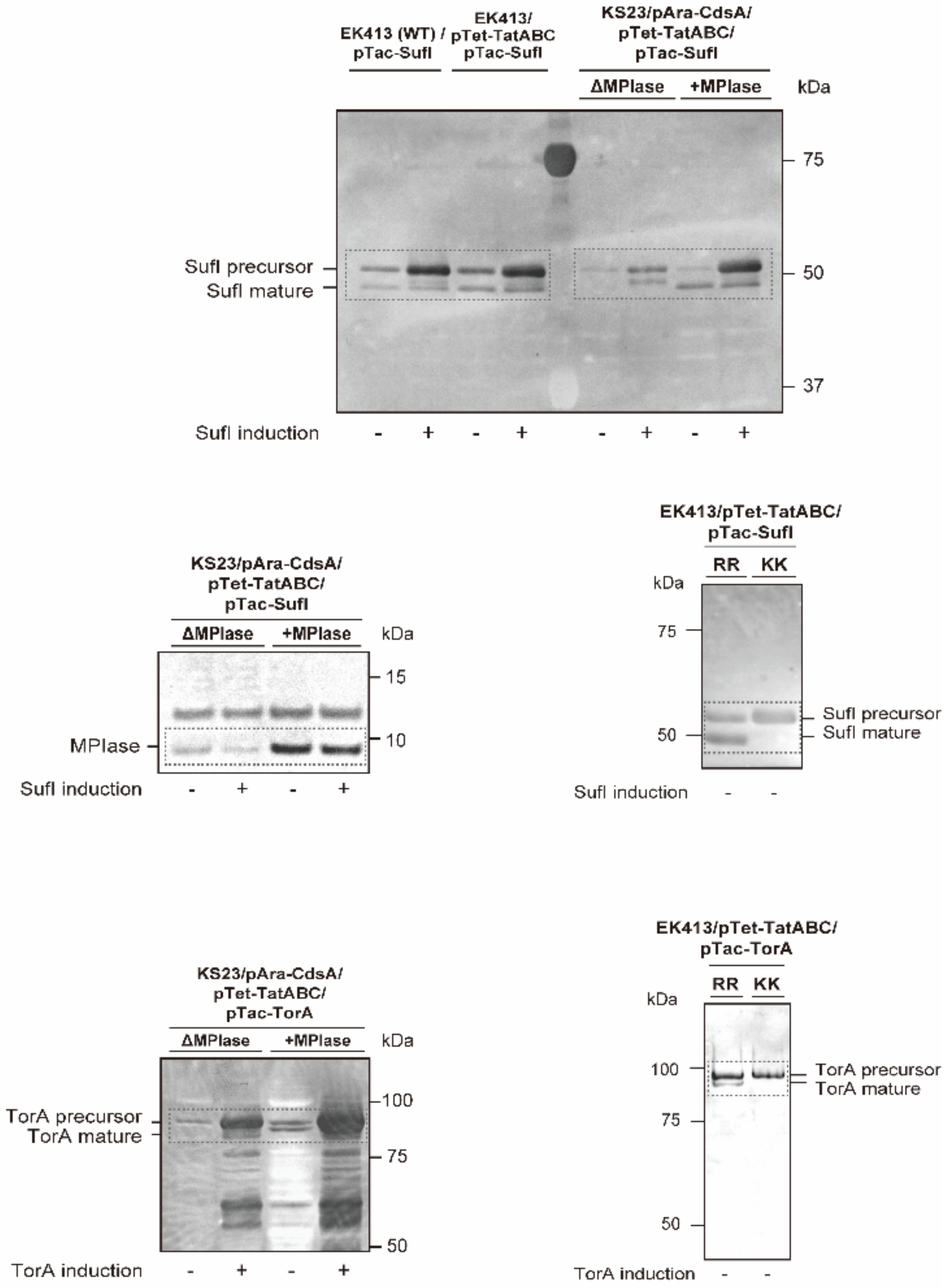
EK413 (WT) / pTac-SufI
EK413/ pTet-TatABC pTac-SufI
KS23/pAra-CdsA/ pTet-TatABC/ pTac-SufI
ΔMPlase
+MPlase
kDa
75
50
37
SufI precursor
SufI mature
SufI induction
- + - + - + - +
KS23/pAra-CdsA/ pTet-TatABC/ pTac-SufI
ΔMPlase
+MPlase
kDa
15
10
MPlase
SufI induction
- + - +
EK413/pTet-TatABC/ pTac-SufI
RR
KK
kDa
75
50
SufI precursor
SufI mature
SufI induction
- -
KS23/pAra-CdsA/ pTet-TatABC/ pTac-TorA
ΔMPlase
+MPlase
kDa
100
75
50
TorA precursor
TorA mature
TorA induction
- + - +
EK413/pTet-TatABC/ pTac-TorA
RR
KK
kDa
100
75
50
TorA precursor
TorA mature
TorA induction
- -

Fig.1B

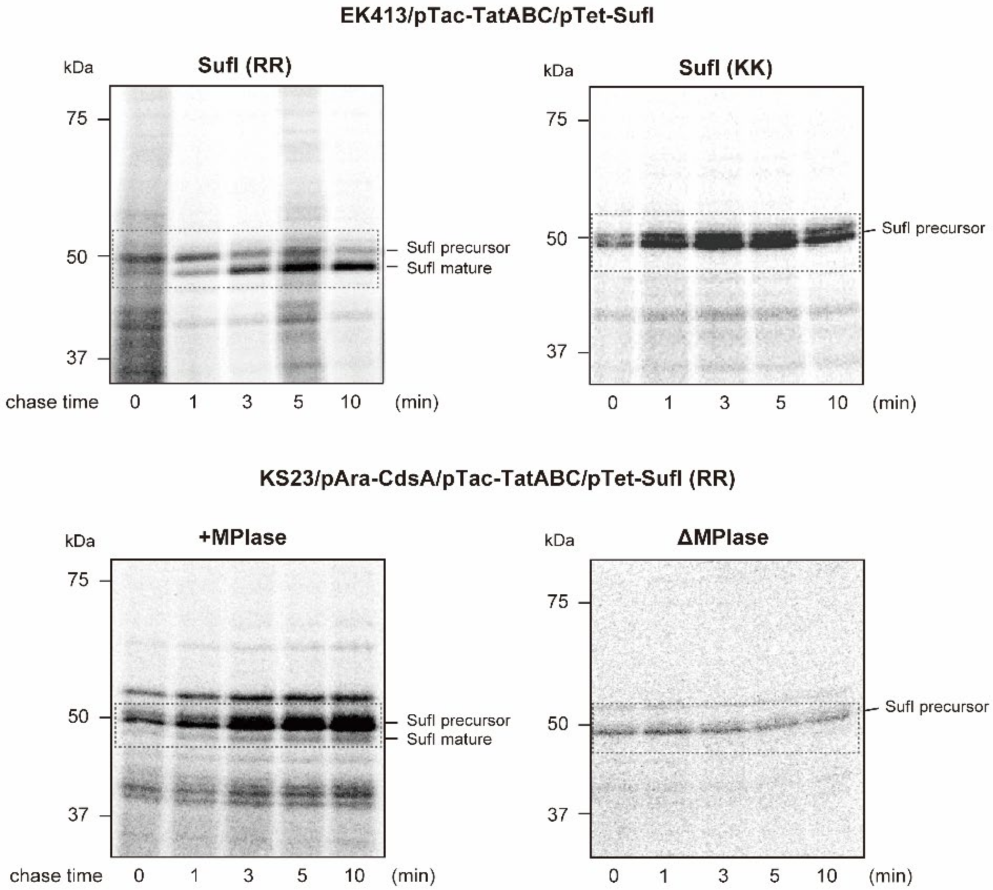
EK413/pTac-TatABC/pTet-SufI
SufI (RR)
kDa
75
50
37
SufI precursor
SufI mature
chase time 0 1 3 5 10 (min)
SufI (KK)
kDa
75
50
37
SufI precursor
0 1 3 5 10 (min)
KS23/pAra-CdsA/pTac-TatABC/pTet-SufI (RR)
+MPIase
kDa
75
50
37
SufI precursor
SufI mature
chase time 0 1 3 5 10 (min)
ΔMPIase
kDa
75
50
37
SufI precursor
0 1 3 5 10 (min)

Fig.1C

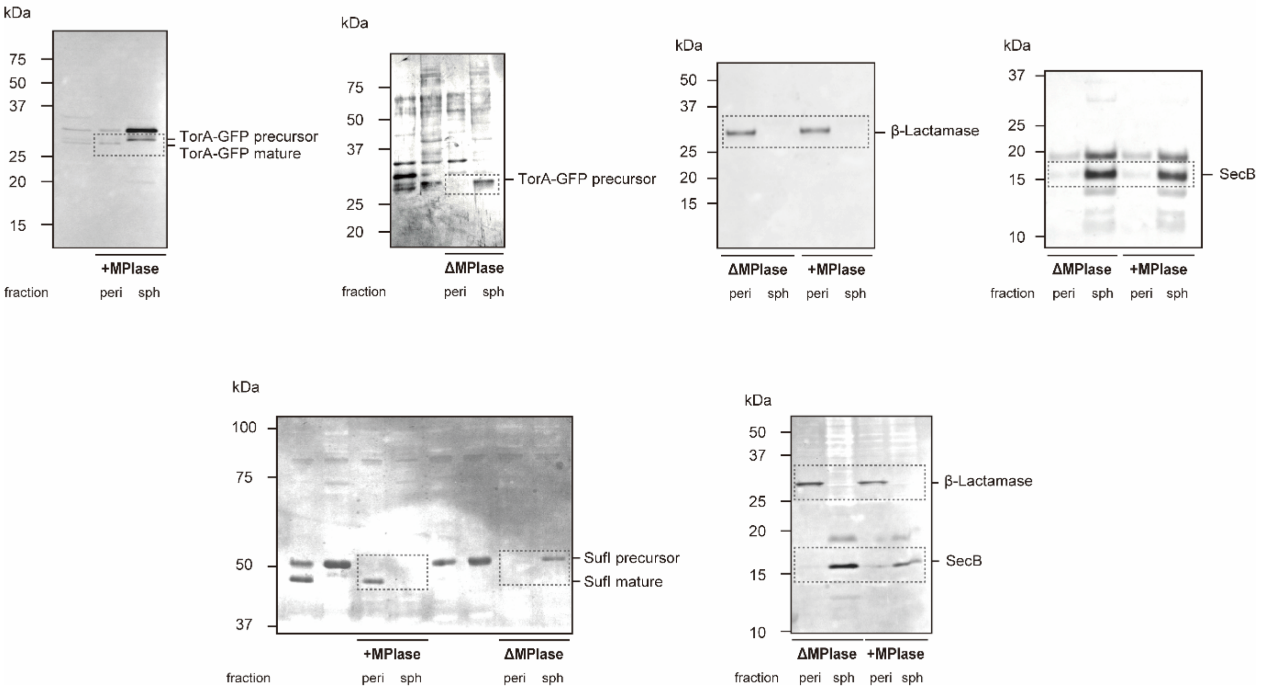
kDa
75
50
37
25
20
15
TorA-GFP precursor
TorA-GFP mature
+MPlase
fraction
peri
sph
ΔMPlase
TorA-GFP precursor
β-Lactamase
SecB
SufI precursor
SufI mature
100
