# Supplementary material for: Glycolipid MPIase is essential for the TAT (twin-arginine translocation) pathway
Source: eLife. 2026 Nov 2;15:RP112170. doi: 10.7554/eLife.112170 (PMC13727073; doi:10.7554/eLife.112170)

Figure 1-figure supplement 2

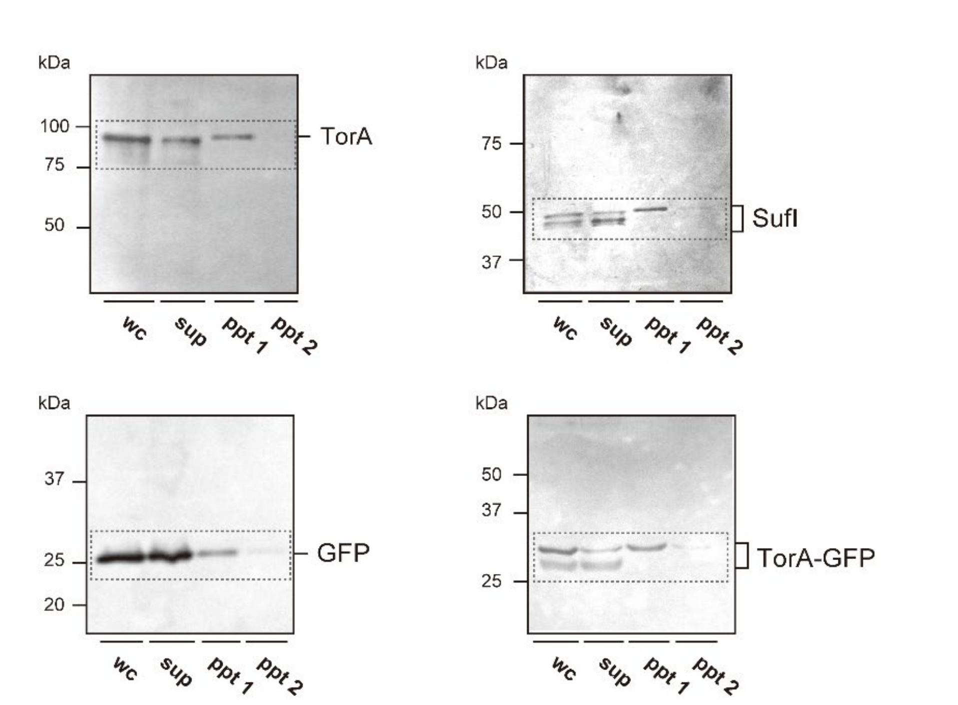

Supplement: Supplementary file 3 [file elife-112170-fig1-figsupp2-data1.zip › Figure 1-source data 3/Figure 1-source data 3.pdf]
