# Supplementary material for: Glycolipid MPIase is essential for the TAT (twin-arginine translocation) pathway
Source: eLife. 2026 Nov 2;15:RP112170. doi: 10.7554/eLife.112170 (PMC13727073; doi:10.7554/eLife.112170)

Figure 2-figure supplement 3

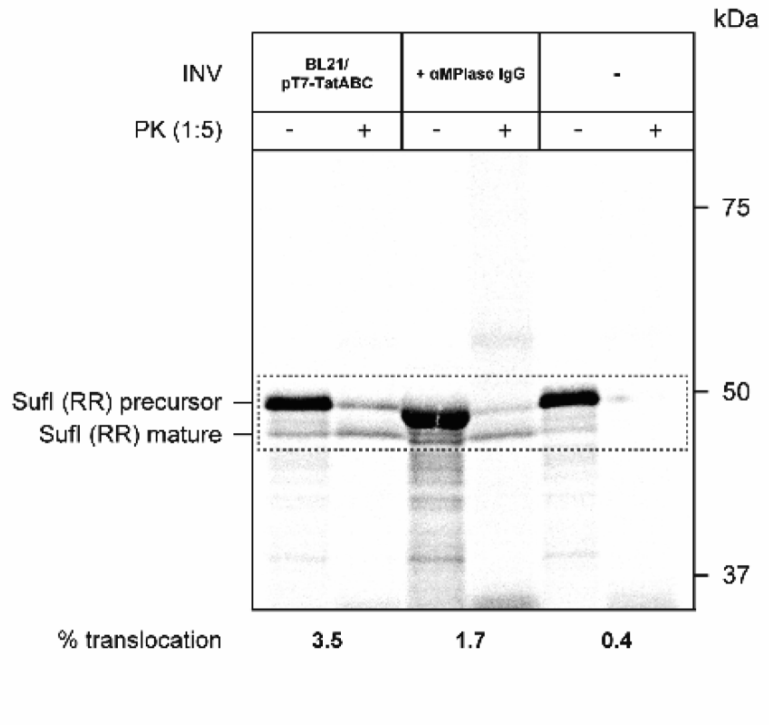

Supplement: Supplementary file 9 [file elife-112170-fig2-figsupp3-data1.zip › Figure 2-source data 5/Figure 2-source data 5.pdf]
