# Supplementary material for: Glycolipid MPIase is essential for the TAT (twin-arginine translocation) pathway
Source: eLife. 2026 Nov 2;15:RP112170. doi: 10.7554/eLife.112170 (PMC13727073; doi:10.7554/eLife.112170)
Supplement: Supplementary file 15 [file elife-112170-fig3-data1.zip › Figure 3-source data 1/Figure 3-source data 1.pdf]

Fig.3A

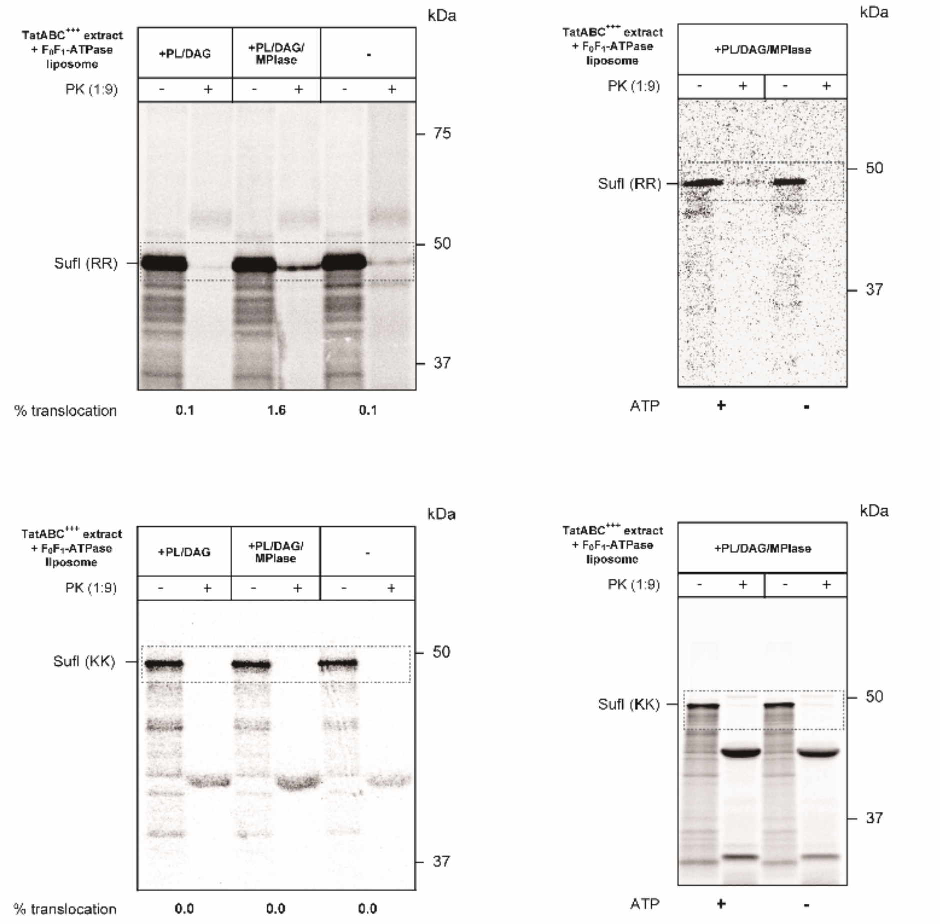
TatABC+++ extract
+ F0F1-ATPase
liposome
+PL/DAG
+PL/DAG/MPIase
-
PK (1:9)
kDa
75
50
37
SufI (RR)
% translocation
0.1
1.6
0.1
ATP
+
-
SufI (KK)
0.0
0.0
0.0

Fig.3C

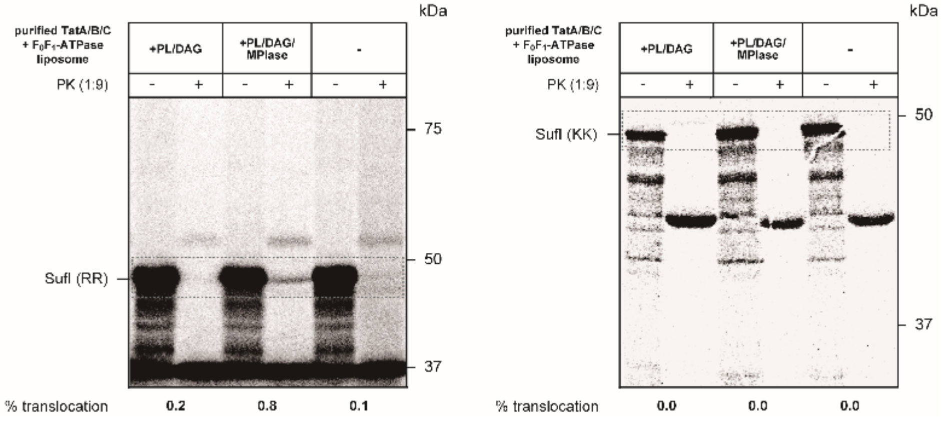
purified TatA/B/C
+ F0F1-ATPase
liposome
+PL/DAG
+PL/DAG/MPIase
-
PK (1:9)
kDa
75
50
37
SufI (RR)
SufI (KK)
% translocation
0.2
0.8
0.1
0.0
0.0
0.0

Fig.3D

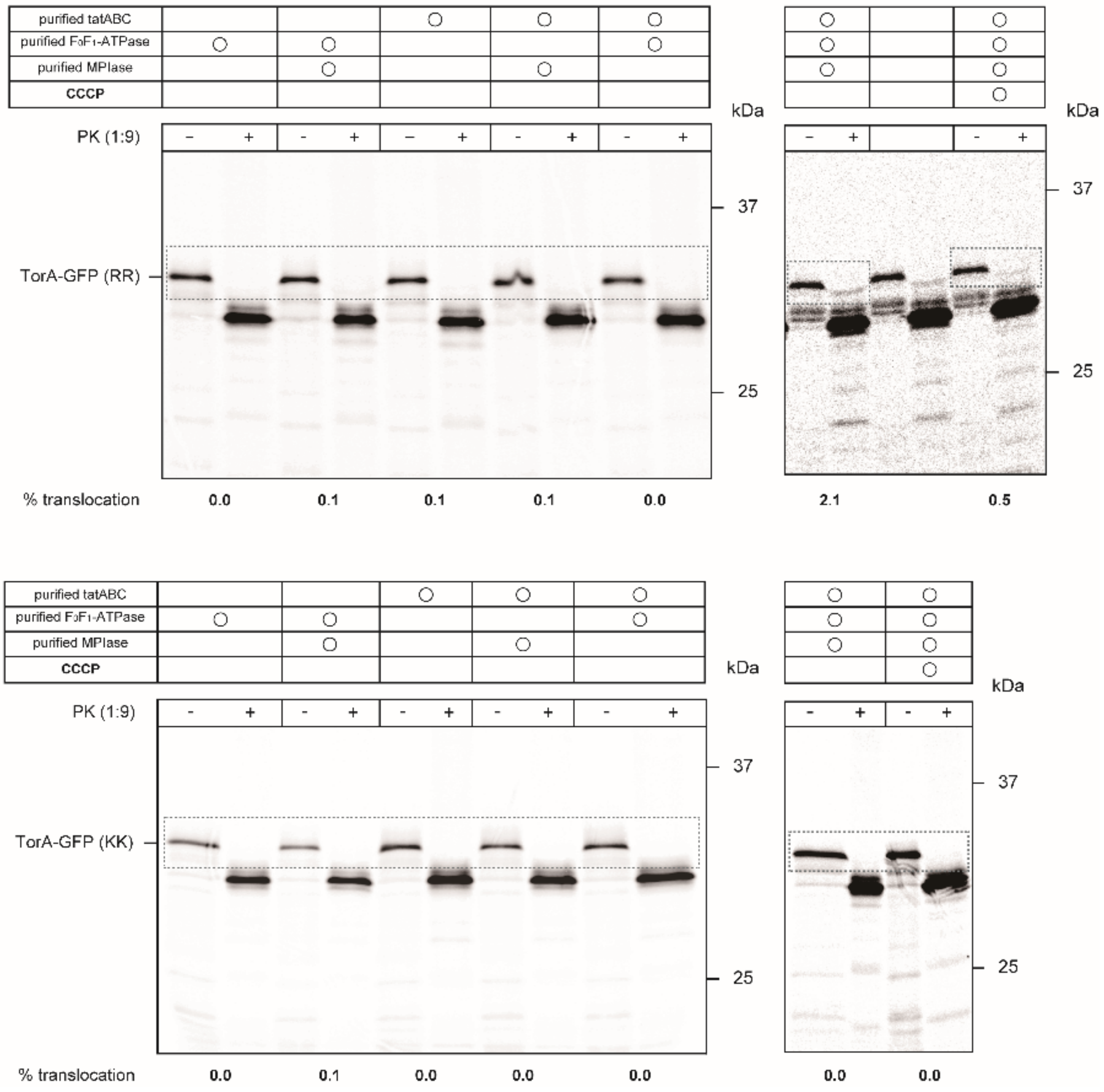
purified tatABC
purified F₀F₁-ATPase
purified MPIase
CCCP
kDa
PK (1:9)
- + - + - + - + - +
37
25
TorA-GFP (RR)
% translocation
0.0 0.1 0.1 0.1 0.0
2.1 0.5
TorA-GFP (KK)
0.0 0.1 0.0 0.0 0.0
0.0 0.0
