# Supplementary material for: Glycolipid MPIase is essential for the TAT (twin-arginine translocation) pathway
Source: eLife. 2026 Nov 2;15:RP112170. doi: 10.7554/eLife.112170 (PMC13727073; doi:10.7554/eLife.112170)

Figure 4-figure supplement 2

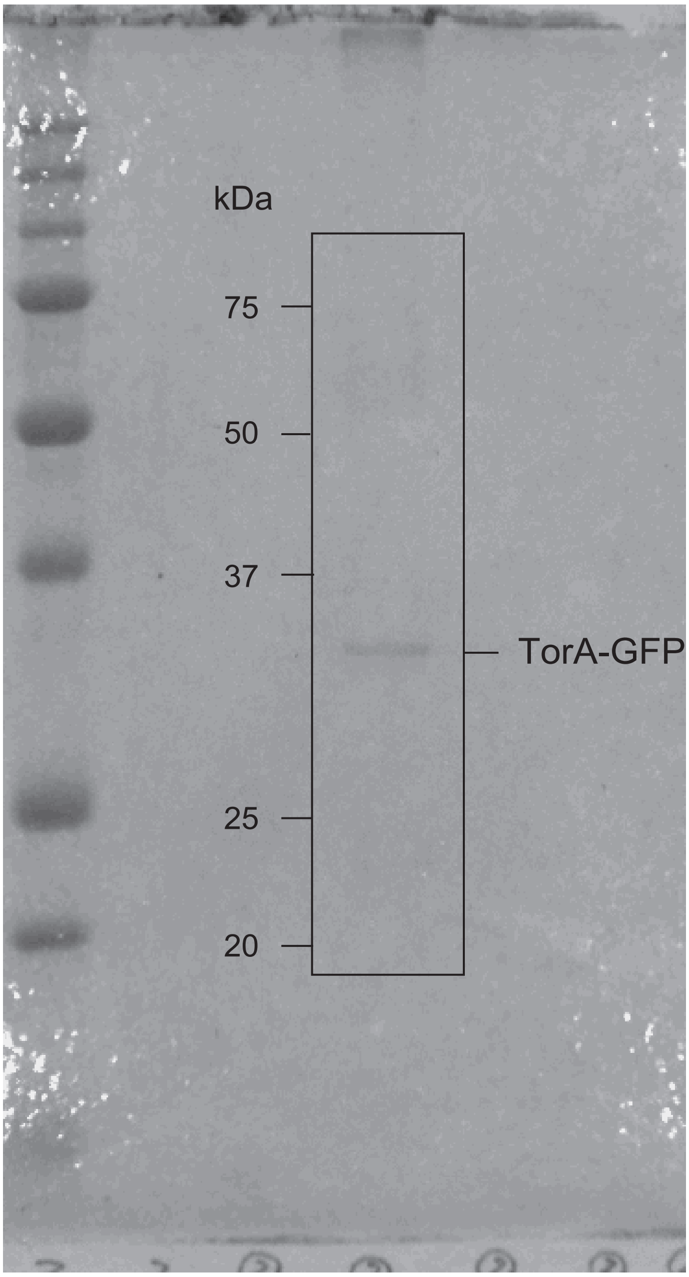

Supplement: Supplementary file 17 [file elife-112170-fig4-figsupp2-data1.zip › Figure 4-source data 2/Figure 4-source data 2.pdf]
