# Supplementary material for: Glycolipid MPIase is essential for the TAT (twin-arginine translocation) pathway
Source: eLife. 2026 Nov 2;15:RP112170. doi: 10.7554/eLife.112170 (PMC13727073; doi:10.7554/eLife.112170)
Supplement: Peer review history

# Editor report: eLife Assessment

**DOI:** 10.7554/eLife.112170.4.sa0

**Authors:** Heedeok Hong

**Affiliations:** Michigan State University United States

**Keywords:** Fundamental, Solid

This study demonstrates a critical role of the glycolipid membrane protein insertase (MPIase) in the twin-arginine translocation (TAT) pathway, a protein translocation system that is conserved across all domains of life. The successful reconstitution of the bacterial TAT system in both bacteria-derived and artificial liposomes provides **solid** experimental evidence that MPIase has a broader role in membrane protein translocation than previously recognized. By revealing the essential function of a nonproteinaceous membrane component in catalyzing a core cellular process, this work offers **fundamental** insights into the molecular mechanisms of protein translocation.

# Referee report: Reviewer #2 (Public review)

**DOI:** 10.7554/eLife.112170.4.sa1

Summary:

In this manuscript, the authors investigated the relationship between the Tat system and MPIase, a glycolipid that facilitates protein integration into the bacterial cell membrane. The TAT (twin-arginine translocation) system is a unique membrane transport machinery that exports fully folded proteins containing a twin-arginine signal peptide. Using both in vivo and in vitro approaches, the authors demonstrated that a sufficient amount of MPIase is required for Tat-dependent protein translocation. Furthermore, the authors successfully reconstituted the Tat transport system by combining recombinant TatA, TatB, TatC, MPIase, and FoF1-ATP synthase.

Strengths:

The reconstituted system clearly demonstrated the requirement for each component, as substrate translocation occurred only when all components were present. Based on these findings, the authors proposed a mechanistic role for MPIase in facilitating Tat-mediated membrane translocation.

Previous studies have shown that MPIase is involved in Sec-dependent protein translocation and membrane protein integration, as well as YidC-dependent membrane insertion. The present study further demonstrated that MPIase also plays an essential role in the Tat translocation pathway.

Overall, this work highlights the central importance of MPIase in bacterial membrane protein biogenesis and provides new insights into the molecular mechanism of Tat-dependent protein transport.

# Author comment: Author response

**DOI:** 10.7554/eLife.112170.4.sa2

**Authors:** Hanako Nishikawa, Nachi Yamamoto, Hayate Kunezaki, Yui Shirogane, Kotoka Kanno, Katsuhiro Sawasato, Miwa Yamada, Ken-ichi Nishiyama

**Affiliations:** Iwate University Morioka Japan; Iwate University Morioka Japan; Iwate University Morioka Japan; Iwate University Morioka Japan; Iwate University Morioka Japan; Iwate University Morioka Japan; Iwate University Morioka Japan; Iwate University Morioka Japan

The following is the authors’ response to the previous reviews.

**Public Reviews:**

[Editors' note: Overall, the reviewing editor and reviewers agreed that authors rigorously addressed reviewers' major scientific concerns.]

**Reviewer #1 (Public review):**

Hanako and colleagues demonstrated that glycolipid MPIase is essential for the TAT system, and they successfully reconstituted the TAT system in vitro for the first time. This will facilitate the understanding of the mechanism of the TAT system.

Comments on revised version from Reviewer 1 and the Reviewing Editor:

In Reviewer 1's points 1 and 2 regarding statistical significance of immunoblotting data (in recommendation to authors), authors stated that they repeated the experiments three times in the rebuttal letter but not in the figure legends. We recommend authors to add a statement such as "errors denote +-SD (N = 3)" in the final version, which will make the data more solid and reliable.

Thank you for the comments. We added the suggested sentence.

**Reviewer #2 (Public review):**

Summary:

In this manuscript, the authors investigated the relationship between the Tat system and MPIase, a glycolipid that facilitates protein integration into the bacterial cell membrane. The TAT (twin-arginine translocation) system is a unique membrane transport machinery that exports fully folded proteins containing a twin-arginine signal peptide. Using both in vivo and in vitro approaches, the authors demonstrated that a sufficient amount of MPIase is required for Tat-dependent protein translocation. Furthermore, the authors successfully reconstituted the Tat transport system by combining recombinant TatA, TatB, TatC, MPIase, and FoF1-ATP synthase.

Strengths:

The reconstituted system clearly demonstrated the requirement for each component, as substrate translocation occurred only when all components were present. Based on these findings, the authors proposed a mechanistic role for MPIase in facilitating Tat-mediated membrane translocation. Previous studies have shown that MPIase is involved in Sec-dependent protein translocation and membrane protein integration, as well as YidC-dependent membrane insertion. The present study further demonstrated that MPIase also plays an essential role in the Tat translocation pathway. Overall, this work highlights the central importance of MPIase in bacterial membrane protein biogenesis and provides new insights into the molecular mechanism of Tat-dependent protein transport.

Comments on revised version from Reviewer 2 and Reviewing Editor:

(Reviewer 2) It is nice to find the mistake in Fig. 4B (RR, EK413, (not +MPIase)) and corrected it in the revised version. However, the data of Fig. 4B and the related modified text on P.9 L256-269 are not very easy to understand what the authors are trying to argue. This reviewer believes that the magnitudes of MPIase expression level are Ek413 > +MPIase > delta-MPIase. Therefore, the means of supernatant in the delta-MPIase and EK413 represents mostly cytosol and periplasm, respectively. On the other hand, the +MPIase shows less supernatant that means a large part of TorA-GFP are trapped nearby membrane but not aggressively transported. Similar results are found also in (KK) case, whereas the sup level in EK413 is lower than that of (RR) case due to the lack of twin-arginine motif in the substrate.

Although these results are convincing in explaining the model in Figure 5, authors might be able to attempt to refine them more understandable. Especially, the revised text on P.9 L256-269 is not enough for leading readers to the author's claim. So that, the authors should revise the text about Fig. 4B again and the related description in the manuscript. Another option is it would be possible to delete whole Fig. 4B, because Fig. 4A, C already well support the model in Fig. 5.

(Reviewing Editor) I agree with Reviewer 2. The first paragraph in the section "MPIase serves as the TAT signal receptor" runs very long and the clarity of the descriptions on Fig. 4B could be further improved. Fig. 4A, C would sufficiently support author's arguments. I recommend that authors consider these options for the final version.

We appreciate the critical comments. In Fig. 4B, we aimed to show that the amount of membrane-targeted TorA-GFP would increase due to the overproduction of MPIase caused by TatABC. At the same time, TorA-GFP translocation was also stimulated. However, since the translocated TorA-GFP was recovered in the same fractions as the cytosolic TorA-GFP, interpreting the results would be complicated. Therefore, we deleted Fig. 4B, as suggested by the reviewer and editor. The corresponding text and the right-hand side of Fig. S9 were also deleted.
